# Understanding the bigger picture: syndemic interactions of the immigrant and refugee context with the lived experience of diabetes and obesity

**DOI:** 10.1186/s12889-021-12305-3

**Published:** 2022-02-18

**Authors:** Nicole Naadu Ofosu, Thea Luig, Yvonne Chiu, Naureen Mumtaz, Roseanne O. Yeung, Karen K. Lee, Nancy Wang, Nasreen Omar, Lydia Yip, Sarah Aleba, Kiki Maragang, Mulki Ali, Irene Dormitorio, Denise Campbell-Scherer

**Affiliations:** 1 Department of Family Medicine, Faculty of Medicine & Dentistry, University of Alberta, Edmonton, Canada; 2 Physician Learning Program, Faculty of Medicine & Dentistry, University of Alberta, Edmonton, Canada; 3 Multicultural Health Brokers Cooperative, Edmonton, Canada; 4 Faculty of Skills and Foundational Learning, NorQuest College, Edmonton, Canada; 5 Division of Endocrinology & Metabolism, Faculty of Medicine & Dentistry, University of Alberta, Edmonton, Canada; 6 Alberta Diabetes Institute, University of Alberta, Edmonton, Canada; 7 Division of Preventive Medicine, Department of Medicine, Faculty of Medicine & Dentistry, University of Alberta, Edmonton, Canada

**Keywords:** Diabetes, Obesity, Migrants, Refugees, Delivery of healthcare, Community health workers

## Abstract

**Background:**

Providing contextually appropriate care and interventions for people with diabetes and/or obesity in vulnerable situations within ethnocultural newcomer communities presents significant challenges. Because of the added complexities of the refugee and immigrant context, a deep understanding of their realities is needed. Syndemic theory sheds light on the synergistic nature of stressors, chronic diseases and environmental impact on immigrant and refugee populations living in vulnerable conditions. We used a syndemic perspective to examine how the migrant ethnocultural context impacts the experience of living with obesity and/or diabetes, to identify challenges in their experience with healthcare.

**Methods:**

This qualitative participatory research collaborated with community health workers from the Multicultural Health Brokers Cooperative of Edmonton, Alberta. Study participants were people living with diabetes and/or obesity from diverse ethnocultural communities in Edmonton and the brokers who work with these communities. We conducted 3 focus groups (two groups of 8 and one of 13 participants) and 22 individual interviews (13 community members and 9 brokers). The majority of participants had type 2 diabetes and 4 had obesity. We conducted a thematic analysis to explore the interactions of people’s living conditions with experiences of: 1) diabetes and obesity; and 2) healthcare and resources for well-being.

**Results:**

The synergistic effects of pre- and post-immigration stressors, including lack of social network cultural distance, and poverty present an added burden to migrants’ lived experience of diabetes/obesity. People need to first navigate the challenges of immigration and settling into a new environment in order to have capacity to manage their chronic diseases. Diabetes and obesity care is enhanced by the supportive role of the brokers, and healthcare providers who have an awareness of and consideration for the contextual influences on patients’ health.

**Conclusions:**

The syndemic effects of the socio-cultural context of migrants creates an additional burden for managing the complexities of diabetes and obesity that can result in inadequate healthcare and worsened health outcomes. Consequently, care for people with diabetes and/or obesity from vulnerable immigrant and refugee situations should include a holistic approach where there is an awareness of and consideration for their context.

## Introduction

Type 2 diabetes and obesity are complex chronic diseases with multiple associated comorbidities, requiring multifaceted approaches to treatment [1, 2]. Personalized healthcare strategies are recommended as they provide opportunity for person-centered approaches to care and to address the complex conditions and drivers related to health [3, 4]. Such strategies provide patients with a sense of agency about their healthcare.

Providing contextually appropriate care and interventions for people with diabetes and/or obesity in vulnerable situations within ethnocultural newcomer communities poses significant challenges. This is because the refugee and immigrant context presents an added complexity to chronic disease prevention and management [5, 6]. Immigrants and refugees bring diverse backgrounds, which significantly impact their lives in the destination country. Although not all migrants are in vulnerable situations, those who are face multidimensional hurdles to securing stable income and obtaining healthcare and supports, which contribute to increased risks of developing acute and chronic health conditions [7]. The entangled complexities of the migrant context and chronic disease necessitate a deep understanding of these interactions to develop appropriate person-centred care.

This study was developed in collaboration with the Multicultural Health Brokers (MCHB) Cooperative of Edmonton, Alberta. The MCHB is operated by community health workers of immigrant and refugee backgrounds, referred to as brokers, who serve 27 ethnocultural communities comprising over 10,000 people speaking 54 languages [8]. Our team included brokers from seven ethnocultural communities, namely Chinese, Eritrean, Filipino, French-speaking African, Somali, South Asian, and South Sudanese communities, who had identified concerns about diabetes and obesity within their communities. They also indicated a missing link in understanding of the interactions between migrant context and illness. We therefore drew on syndemic theory [9, 10] and the population health approach [11] to capture determinants of health, and synergistic effects of health, illness experience and vulnerable conditions. Syndemic theory is a population health theory, which sheds light on the synergistic nature of stressors, chronic diseases and environmental impact on immigrant and refugee populations living in vulnerable conditions. The key contribution of syndemics is its attention to the large-scale social forces that give rise to higher concentrations of disease and worse outcomes in certain populations, explaining how sociocultural, historical, and geographical realities interact with and compound the adverse consequences of disease [12]. This study uses a syndemic perspective [9, 10] to examine how the lived experiences of migration and rebuilding a life as part of an ethnocultural community impacts the experience of living with diabetes and obesity, to identify challenges in the healthcare experience.

## Methodology

This qualitative study is part of a multi-sectoral research project, using a multi-method, collaborative, participatory approach [13, 14] to identify care gaps in obesity and diabetes prevention and management in vulnerable newcomer ethnocultural communities. We relied on the expertise and engagement of the brokers to guide the data collection design, participant recruitment and analysis. We held monthly community advisory group meetings with the brokers over the course of the project.

### Participants, data collection and analysis

Adult community members with diabetes and/or obesity from the seven ethnocultural communities were eligible to participate in the study. The brokers from each participating ethnocultural community informed eligible community members of the study. Interested participants provided contact information that the broker passed on to the research team to follow up for informed consent to participate. Where necessary, brokers provided translation between the research team and community members. The brokers also participated in data collection by providing background information on their respective communities, insights on their own experiences with diabetes and/or obesity if they had these chronic diseases, and interpretation for community members with limited English language ability. All participants including the brokers provided signed written consent. This research with human subjects was done with informed consent and in accordance with the declaration of Helsinki. The University of Alberta ethics board (Pro00089571) approved this study.

We generated data through focus groups and interviews. Community participants received grocery gift cards after the interviews or focus groups. The brokers also received grocery gift cards for their role in data collection and the community advisory group. We used semi-structured interview and focus group guides for data generation. Questions were open-ended and participants were encouraged to share experiences freely. Additional questions were added during interviews to deepen and clarify responses. The focus phenomenon was the lived experience of obesity and/or diabetes for people from ethnocultural communities, exploring: 1) conditions in their lives that impact their experience; and 2) experiences of healthcare and resources for well-being.

Data collection and analysis were iterative. The researchers conducted all the interviews and focus groups in English but where participants could not communicate in English, the broker for their community provided translation for the researcher and participant. For the focus groups, there was one broker per community represented. Interviews and focus groups were audio recorded and transcribed verbatim in English. Data were managed and coded in NVivo (QSR International Pty Ltd. Version 12, 2018). We conducted a thematic analysis of the data, an iterative process of immersion in data, inductive development of codes, coding, comparing coded data within and between data sources to identify patterns of meaning, generating and refining themes from the patterns in relation to the research question [15, 16]. We used an interpretive qualitative approach, describing the meanings people attribute to the phenomenon [17, 18], while keeping descriptions at a level stakeholders can relate to so that they can better understand the situation and motivate action [19]. Data were first cross-coded by NNO, TL, NM & DCS and a code manual was collaboratively developed based on discussions from the cross-coding. NNO and NM re-coded the data using the code manual. Patterns in the data were identified and discussed with the research team and community partners (the MCHB). In collaboration, these patterns were further abstracted to generate themes and themes were refined to illuminate meaning behind data around a central concept (i.e. impact of migration, financial constraints, cultural distance). Field notes from data collection were referred to during the analysis for additional contextual information. We recognize our participants as co-researchers and the understanding achieved in this work as co-created knowledge.

### Rigour

We incorporated processes to ensure trustworthiness of this research [20, 21]. Our participatory approach, involving regular discussions and documentation (audit trail) of the research process with our community partners, helped ensure credibility and dependability of our findings. Method, data generation strategy, sample size, data analysis processes were all methodologically aligned with our research question to establish methodological coherence [20, 21].

## Results

We conducted three focus groups comprising two groups of eight mixed male and female participants and one group of 13 female participants. We also conducted 22 individual interviews with five male and eight female community members and nine female brokers (Table 1). Collaboratively, we abstracted four themes from the data: 1) Impact of immigration status and pathway on health; 2) Post immigration stressors affect people’s capacity to prevent or manage diabetes and/or obesity; 3) Socio-cultural context of refugees and immigrants and, disease and treatment burden; and 4) The nature of communication and relationships with providers affect how patients experienced care.

**Table 1:** Study participant description

Community	Gender & health condition (obesity/diabetes) or n/a – not applicable	Interpreter present? Yes/No (Language)
**Interviews**		
1. French-speaking African	Male - obesity	No
2. French-speaking African	Female - obesity	No
3. French-speaking African	Male - diabetes	No
4. French-speaking African	Male - diabetes	No
5. Chinese	Female - diabetes	Yes (Chinese)
6. Eritrean	Female – diabetes and family caregiver	Yes (Tigrinya)
7. Eritrean	Male - diabetes	Yes (Tigrinya)
8. Eritrean	Female - diabetes	No
9. South Sudanese	Male - diabetes	No
10. South Sudanese	Female - diabetes	No
11. South Sudanese	Female - diabetes	No
12. South Sudanese	Female - diabetes	Yes
13. South Sudanese (broker)	Female – diabetes & obesity	No
14. South Sudanese (broker)	Female - diabetes	No
15. Somali	Female – diabetes & obesity	Yes (Somali)
16. Chinese (broker)	Female - diabetes	No
17. Eritrean (broker)	Female – n/a	No
18. Filipino (broker)	Female – n/a	No
19. French-speaking African (broker)	Female – n/a	No
20. Somali (broker)	Female – n/a	No
21. South Asian (broker)	Female – n/a	No
22. South Sudanese (broker)	Female – n/a	No
**Focus group discussions**		
1. South Asian	Males – 2; Females – 6 (diabetes)	Yes
2. Chinese (4) and Filipino (4)	Male – 1; Females – 7 (diabetes)	Yes
3. Somali	Females −13 (diabetes)	Yes

Table 2 summarizes information from interviews with the brokers on the participating ethnocultural communities that contributes to their socio-cultural context. These realities present multidimensional challenges to understanding Canadian culture and systems, developing language skills, education and training, and addressing parenting challenges. They subsequently contribute to the context affecting people’s capacity to manage their health conditions.

**Table 2:** Summary background information of ethnocultural communities participating in this study

**Chinese**	
**Countries or regions represented:** Chinese speaking people from all areas including mainland China, Hong Kong, Macau, Taiwan, Malaysia, and Singapore.	
**Predominant immigration pathway or status:** Family sponsorship (for seniors, spouses), Skilled worker, landed immigrant/permanent resident, temporary foreign worker, visitor, and student.	
**Pre-immigration realities:** These are usually people who have self-elected to move to Canada for a better life for themselves and their families. There are a mixture of people of different backgrounds in terms of education/training, and age. They usually have strong family ties.	
**Some key post-immigration realities**: ∙ Language barrier (especially for seniors) ∙ Difficulties parenting in two cultures – because the Chinese ways of raising a child may be different in some ways from the Canadian ways. ∙ In moving to Canada on sponsorship of their children, seniors tend to lose their traditional role of head of family with a lot of influence on happenings in the family. They usually tend to be dependent on their children. This sometimes leads to family dispute situations.	
**Eritrean**	
**Countries or regions represented:** All areas of Eritrea.	
**Predominant immigration pathway or status:** Primarily refugees who have lived in countries neighbouring Eritrea for several years, family sponsorship, and visitors who later change to refugee claimants.	
**Pre-immigration realities:** Eritrea has faced a lot of political instability, so people coming to Canada from this background have faced the trauma of war and the harsh realities of life as refugees in other countries. This background also affects the family systems as it leads to families being divided which in turn impacts the socialization process of younger generations into what typical family life looks like, and how parenting is done. Additionally, people may have difficulty accessing appropriate formal education in refugee situations. Given poor living conditions such as inadequate food, medication, and shelter, people’s health are adversely impacted.	
**Some key post-immigration realities**: ∙ Domestic violence situations ∙ Difficulties parenting in two cultures ∙ Language barrier / Inadequate education or training	
**Filipino**	
**Countries or regions represented:** All areas of the Philippines. Additionally, people immigrate into Canada from other countries like Hong Kong, Singapore where they live and work.	
**Predominant immigration pathway or status:** Landed immigrant/permanent resident, live-in caregiver, family sponsorship, temporary foreign worker, and visitor.	
**Pre-immigration realities:** These are usually people who have self-elected to move to Canada for a better life for themselves and their families. Usually well-educated, professionals with strong family ties.	
**Some key post-immigration realities**: ∙ The skills and training for which they gained adequate points for immigration are usually not recognized when they migrate to Canada because they are foreign trained. This may cause difficulties securing certain kinds of jobs and affect the settling down process. ∙ Work situations in which parents have multiple jobs so do not have much time for their children or their own health. ∙ Temporary foreign workers are confined by their boundaries of their contract. Opportunities for renewal and subsequently a path to being an immigrant can be challenging. ∙ Difficulties parenting in two cultures. Sometimes leading to cases with Children's Services.	
**French-speaking African Community**	
**Countries or regions represented:** French-speaking countries in Africa	
**Predominant immigration pathway or status:** Refugees, skilled workers, landed immigrants/permanent residents, foreign workers (being on work permits)	
**Pre-immigration realities:** Those from countries where there has been civil war, for example, DR Congo and Burundi, have experienced trauma from the war and life as refugees in neighbouring countries. There are a lot of people separated from their families and this impacts the socialization process of younger generations into what typical family life looks like, and how parenting is done. Additionally, people are unable to access appropriate formal education in the refugee camps. Given poor living conditions such as inadequate food, medication, and shelter, people’s health are adversely impacted. Those that come in as skilled workers are usually people who have self-elected to move to Canada for a better life for themselves and their families. They usually are well-educated.	
**Some key post-immigration realities**: (mainly faced by refugees) ∙ Poverty and low-income situations (low income/government housing, and dependence on social support services). ∙ Large families unable to afford adequate housing space so they have to live in cramped living conditions. ∙ Language barrier. ∙ Difficulties acquiring education or training for meaningful employment (because of language barrier, income, family situation, no previous history with the formal education system) ∙ Parenting challenges arising from impact of refugee situation on socialization into family life; and difficulties parenting in two cultures. Issues with Children's Services may arise from these conditions. ∙ Children struggle to fit into the school system as it is unfamiliar and also because they feel ‘othered’. Parents also struggle to help their children with their school work since they may not have had much education themselves. ∙ For the skilled worker or foreign worker, the skills and training for which they gained adequate points for immigration are usually not recognized when they migrate to Canada because they are foreign trained. This may cause difficulties securing certain kinds of jobs and affect the settling down process.	
**Somali**	
**Countries or regions represented:** All regions of Somalia	
**Predominant immigration pathway or status:** Government sponsored refugees	
**Pre-immigration realities:** Somalia has had civil war since the late 1990s, most Somalis have lived as refugees in neighbouring countries prior to coming to Canada. Those who immigrated prior to the 90s came mainly on scholarships for education purposes and ended up becoming permanent residents and citizens of Canada. Those who came after the start of the civil war have since come as refugees. People have experienced trauma from the war and many years of life in refugee camp settings. There are a lot of people separated from their families and this impacts the socialization process of younger generations into what typical family life looks like, and how parenting is done. Additionally, people are unable to access appropriate formal education in the refugee camps. Given poor living conditions such as inadequate food, medication, and shelter, people’s health are adversely impacted.	
**Some key post-immigration realities**: ∙ Poverty and low-income situations (low income/government housing, dependence on social support services) ∙ Large families unable to afford adequate housing space so they live in cramped living conditions ∙ Language barrier ∙ Difficulties acquiring education or training for meaningful employment (because of language, income, family situation, no previous history with the formal education system) ∙ Parenting challenges arising from impact of refugee situation on socialization on family life; and difficulties parenting in two cultures. Issues with Children's Services may arise from these conditions. ∙ Domestic violence situations may also come up with changing family role dynamics where the father may lose his role as breadwinner. ∙ Children struggle to fit into the school system as it is unfamiliar and also because they feel ‘othered’. Parents struggle to help their children with their school work since they may have not had much education themselves.	
**South Asian Community**	
**Countries or regions represented:** India, Pakistan, Bangladesh, Nepal, Sri Lanka, Bhutan	
**Predominant immigration pathway or status:** Landed immigrants, family sponsored immigrants	
**Pre-immigration realities:** These are usually people who have self-elected to move to Canada for a better life for themselves and their families. Usually well-educated with strong family ties.	
**Some key post-immigration realities**: ∙ The skills and training for which they gained adequate points for immigration are usually not recognized when they migrate to Canada because they are foreign-trained. This may cause difficulties securing jobs commensurate with their training and affect the settling down process. ∙ Being a visible minority in some places, they sometimes face discrimination. ∙ Difficulties parenting in two cultures. ∙ Issues with family violence - one of the causes being changes in power dynamics and gender roles. For example, women having opportunities to work and earn, whereas the man may be struggling with finding a stable job.	
**South Sudanese Community**	
**Countries or regions represented:** Families come from all regions of South Sudan.	
**Predominant immigration pathway or status:** Government-sponsored refugees	
**Pre-immigration realities:** South Sudan had been war-torn for over fifty years, so many South Sudanese people coming into Canada have lived for many years as refugees in refugee camps in neighbouring countries prior to coming. People have trauma from the war and refugee situation. There are a lot of people separated from their families and this impacts the socialization process of younger generations into what typical family life looks like, and how parenting is done. Additionally, people are unable to access appropriate formal education in the refugee camps. Given poor living conditions such as inadequate food, medication, and shelter, people’s health are adversely impacted.	
**Some key post-immigration realities**: ∙ Poverty and low-income situations (low income/government housing, dependence on social support services) ∙ Language barrier ∙ Difficulties acquiring education or training for meaningful employment (because of language, income, family situation, no previous history with the formal education system) ∙ Parenting challenges arising from impact of refugee situation on socialization on family life; and difficulties parenting in two cultures. ∙ Children struggle to fit into the school system as it is unfamiliar and also because they feel ‘othered’. Parents struggle to help their children with their school work since they may have not had much education themselves.	

### Impact of immigration status and pathway on health

There are diverse immigration pathways and status by which people come to Canada, such as refugee, skilled worker, family sponsorship, caregiver, temporary resident and student. As indicated in Table 2, these pathways and status encompass the origin and experience of the different migrant populations, and the scope and nature of the immigration process. Consequently, they have implications for the health characteristics with which people present to Canada and the kinds of resources people are aware of or can access to support their health needs. Most of Canada’s immigration pathways select healthy immigrants, however, given that the resettlement process comes with challenges, knowledge of and access to settlement supports is crucial to immigrants’ continued health and wellbeing. As illustrated in the quote below, migrants desire such information and support to manage their lives as newcomers to keep healthy.

*“Maybe someone will be there to explain to us as a newcomer, ‘Okay, here is no more sunny place, it’s a cold place …change your habit’. But if nobody talks about that or teach you, how will you understand? They can explain at the beginning everything to the newcomers about health, about paying attention to their body and everything. [For instance], because of the weather, you need to change your habit or you will gain weight or get high blood pressure because of this. This kind of information will help people to be in good condition and, it is going to help the government because the government don’t spend money for their health. …About my condition, I came here six years without sickness, but when I enter in seven years I start to realize something is wrong. …my health condition going down. If someone had been there to warn me to say, ‘Be careful what you eat, be careful what you are doing’, that would have been helpful to keep me [healthy] longer.” (I1)*

The refugee pathway usually does not have a stringent healthy refugee requirement because of the humanitarian situation. Thus, refugees may present with some trauma, physical and mental health issues because of disturbing situations from their country of origin. Government-sponsored refugees go through a health program upon arrival that links them with family doctors with coverage for 1 year. Although limited, this program provides them with some awareness of the healthcare system.

*“So there is a one-year program for newcomers, and that is specifically for refugees. There’s the newcomer clinic through Catholic Social Services, and the clinic is at East Edmonton Community Health Centre. There is an interpreter there, so for that first year, families that come into the country as refugees – and I need to clarify whether if family members sponsor refugees, if that falls under [the program]. But for sure, government sponsored, I know that is there. For that one year there’s interpretation available, you will see the dietician if need be, you have the doctors that you will see in that clinic. So there’s more help available for the very very newcomers, within that one year. But after that one year, you go on your own.” (Focus group 3_P13)*

Participants felt that the lack of warm hand-off to supportive services following this program could be detrimental to the process of settlement since people leaving these programs may not be adequately prepared to navigate the new environment. Migrants from the other immigration pathways may also have difficulty navigating available health services without informative processes about services and resources available to them to care for their health. These situations contribute to missed opportunities for interventions for diabetes and obesity prevention and management.

Immigration pathway and status are therefore important determinants of health. The interaction of migration pathway and consequently different public services that affect people’s knowledge of and access to resources and supports for settlement can leave people vulnerable to settlement stressors that can affect their wellbeing.

### Post immigration stressors affect people’s capacity to prevent or manage diabetes and/or obesity

Post immigration challenges synergistically interact to generate stress in people’s lives. We identified three sub-themes of stressors affecting participants’ experience of diabetes and obesity: 1) financial constraints, 2) lack of social network and 3) cultural distance.

#### Financial constraints

People experience financial constraints because of inadequate opportunities to earn income due to personal or systemic factors. Personal factors include lack of skills for employment such as language, education or training. Although there are training opportunities, people’s situations may render them unable to take up these opportunities. Additionally, many migrants are in situations where they are financially responsible for relatives in their country of origin. Some have children, aging parents, siblings and other relatives they had to care for. This put an additional strain on their financial situation. Systemic barriers arise in situations where policies create barriers for people to overcome poverty. For example, government sponsored refugees are required to pay back the transportation loan for bringing them to Canada. Without repayment, they are unable to apply for citizenship. Some people with skilled worker status face the challenge whereby the very skills or training that enabled them to immigrate not being recognized in Canada. They too must pursue additional education, training and licensing to seek jobs commensurate with their training. These situations along with the immediate pressure to start earning income to support one’s self and family upon arrival in Canada pushes people to pursue low-paying jobs that are more readily available. As illustrated in the quote below people in such situations may feel unduly burdened:

*“P: As I told you before, when we came, for a year we didn't work any job, we didn't find – we searched but we couldn't find. At that time, a little bit harder for us… Again her case of diabetes when she came to Canada here. So like that, we faced some challenges… For now, I'm searching for security guard job and then, maybe, who knows one day I will work with my profession.*

*I: So with your profession, is there an opportunity to write an exam or something?*

*P: ...Yeah. But first they asked me to upgrade my English, and like that. You see, and because of my age, everything is challenging. I want to work, I need to feed my children. It's a little bit harder.” (I6)*

*The charge back home [country of origin] didn’t change. You still pay rent there for brothers and sisters, niece and nephew because they are going to school. You want them to finish their school and make you free because if they don’t succeed in school, that means that’s your load for a lifetime and you want to remove this load to put it somewhere. That means you push them to finish school so that you’ll be free… no more paying their school fees and house rents because they have a job and everything is okay. When you are working on this, your load [in Canada] doesn’t also change. … So, I said okay, I have to stop going to the gym because you don’t want [them] back home to stop the school. You sacrifice yourself. That’s a problem and more immigrants are facing this. (I1)*

Financial constraints impact people’s health in diverse ways, including reduced capacity to prioritize and manage their health and generating stress which takes a toll on people’s health. People in this situation are unable to engage adequately in important health-promoting activities like being able to afford the time and money to cook and eat well, exercise, rest, and go for regular medical check-ups, because of other urgent needs taking up their resources.

#### Lack of social network

Participants felt that having a social network such as family, ethnocultural community groups and religious groups was important for their wellbeing and ability to navigate an unfamiliar environment. Where there had been experiences of lack of social network, people felt impacts on their mental health, worsened stress and decreased capacity to care for their health. It also exacerbated experiences of isolation, especially for people dependent on the welfare system and who lacked language skills to engage meaningfully in their environment. As illustrated in the quotes below, in the absence of a social network people more easily succumb to stress which affects their health.

*“I told you my husband has to work sometimes 20 hours/day, five days and I'm the one who takes care of all my kids and my husband. Sometimes I say, oh, it’s too much for me, but I have to do that because we need to have what we need to live. We have many bills to pay and my husband has lots of things to do… Sometimes, I don't have time to take care of me. I don't have time for me… When I got married [in country of origin], I continued working. My mom was there, my sister and after work I can go to exercise to do my Zumba and come back home. But when I come to Canada I have to stay home with the kids. I cry every day at home. ...It’s difficult for me to accept that and I start to gain weight. Gain weight, because it’s every time chocolate. I tried to find a job, no job. And when I find a job the hours was not good. Because my husband have like scheduled job. He can’t stay at home when I do something outside.” (I2)*

*“I have diabetes. I come Canada for 20 years… First time I got cancer, I live in Canada alone. You know, nobody care for me, nothing. (Focus group 2_ P7)*

Social network is important as it can provide supports to mitigate the health risks associated with the challenges of settlement in a new environment.

#### Cultural distance

Cultural distance refers to differences between Canadian culture and the cultures of newcomers. Cultural distance is observed in various facets of the lives of migrants, including interactions with the healthcare system, parenting in a different culture, gender roles, language barrier, interactions with the legal system, and adapting to the education system.

*“The key challenge is the language barrier. Yeah because some people, even back home, they’re illiterate. They speak the language but they don’t write it. The language barrier is the big thing here for our community, and lack of intervention and lack of awareness is a big thing, and parenting is very difficult.” (Focus group 3_ P13)*

*“She was seeing a doctor when she first arrived and she wasn’t feeling well, and... when she said a symptom and he will ask when did it start? And being from refugee camp, she will tell me, like when I had my son. For me, as a broker, I have to figure [the timeframe] out. So I had to go back to her and explain to her, like so when was that? And then I will ask her when was he born? And then I will do calculation and kind of figure. The doctor goes and says can you make a long story short?” (I15)*

As illustrated by the quotes above, cultural distance can pose significant challenges to one’s ability to settle and function meaningfully in society. Additionally, in the context of healthcare, cultural distance presents a barrier to communication between patients and healthcare providers. It can reduce opportunities to employ the personalized care strategies that are relevant to obesity and diabetes management.

### Impact of migrant context on disease and treatment burden

As illustrated above, the post migration experience involves unique challenges including less opportunity to earn adequate income, cultural distance, and the loss of a social support network. Within this context, participants described their experience of living with diabetes and obesity. Theyexpressed that living with diabetes and/or obesity created an added burden on themselves and their families because of the indirect and direct treatment costs. Examples include challenges maintaining a regular supply of medication and blood sugar testing supplies, and changing one’s diet with an already strained family budget. The hurdles and stressors of people’s contexts also creates limited capacity for lifestyle changes that are important for diabetes/obesity management. These changes may be difficult to accommodate when families have limited financial and time capacity for changes such as special diets, extra shopping and cooking. Maintaining regular physical activity was also challenging given weather conditions and built environments that do not support active transportation. Consequently, in the face of competing priorities arising from their socio-cultural context, people felt pressured to sacrifice their own health to accommodate other needs.

*They tell me to buy diabetes supply… So I go to the pharmacy and they say it costs me about 90 bucks and bring like that…..No coverage. I have to pay... But that time I don’t got money in my pocket, so I say I have to wait until I get the pension coming. I tell them (Focus Group 2_P3)*

*“I’ll say, it’s hard to change the food. Because now, I’m watching everything I’m eating and that means double cooking. Yeah, because they [family] like something that I used to like but now, I can’t eat it because I’m watching everything I’m eating… It costs a lot. Sometimes I have to say, ‘Okay, eat and I’m okay for today’, but I’m not okay, but I have to say in front of them, I’m okay, yeah, to make them comfortable to eat. And I laugh with them and I make a joke. I don’t want to show them I want to eat, I want to show them everything is okay.” (I1)*

The challenges of the migrant socio-cultural context exacerbate the burden of living with diabetes and obesity.

### The nature of communication and relationships with providers affect how patients experienced care

People reported being able to access healthcare, see a physician, and being offered access to allied healthcare providers and programs to support diabetes or obesity management. However, uptake of such services for well-being, beyond the doctor visit was a challenge because of factors like needing language translation, childcare, work hours conflicting with available times of these services, and lack of understanding of services being offered. Additionally, people were not aware of the kinds of primary care services available.

Participants also expressed concerns about perceived side effects of diabetes medications such as weight gain and impotence in men. However, Some participants in these situations did not feel comfortable discussing their concerns with their healthcare providers, they rather sought alternate treatments.

*R: Fenugreek. I use every day, fenugreek and without sugar and it helps me to regulate my blood sugar. …I don’t want insulin. Insulin is sometimes like … it treats my blood sugar if high, but it gains weight a lot... Yeah, it gains a lot and when I started insulin … I did feel like bad things, before I didn’t feel in my head here, and my heart, I didn’t feel like this. Now, when I was started insulin, different feeling. …Yeah, it’s insulin. It helps to lower your blood sugar, but some effect of insulin is like gain weight and I think for kidneys not good. When I use insulin I didn’t feel good.*

*I: So, are you able to discuss this with your doctor, to tell your doctor how you feel about it?*

*R: Maybe I will discuss*

*I: So you haven’t talked to your doctor before?*

*R: Yeah. (I8)*

In situations where people perceived the healthcare provider to be aware of and responsive to their context, it enhanced patient-provider communication and relationship, even when translation was needed. This created the space for asking questions and exchanging knowledge as well as coming to a shared understanding of expectations of each other. It also helped patients to address issues specific to diabetes and weight management by finding options that were realistic within their context. The following quotes illustrate two contrasting perspectives of patient-provider interactions:

*R: Every time I see him [family doctor], no more than 10 minutes. Every time I ask what’s wrong with me, then “Oh okay, okay, I give you…” He just go to typing and he give me the prescription. I have him no more than 10 minutes.*

*I: So you’re unable to ask all your questions?*

*R: No, he write on the wall, only one question [per visit].” (Focus group 2_P3)*

*The doctor is like a father, mother, like brother, so excellent doctor, so so nice. Yeah. When I came to his office, his face is very happy and fifty per cent when I talk to him, my stress is gone. He understand my issues, my feelings, everything, yeah.” (I6)*

Participants felt that having healthcare providers who could work with them within their context and having support to navigate the challenges of their socio-cultural context would enhance their experience of healthcare

*“R: I think that is the way we have to work and the way we can also help our communities [is to] invite the doctors sometimes into the community. They can sometimes give their time in the community too. Yeah, I think that is what we have to do, yeah” (I4)*

Participants also highlighted the role of the brokers as important to mitigating the effects of the socio-cultural challenges they faced. As illustrated in the quotes below, brokers walk alongside families to help address their needs.

*“She helped me with a lot when I came here... I was new and did not know how to manage here in Canada.” (I2)*

*“I 1: Do you still go for the appointments with her?*

*F 4: Well, not now. Now she’s better, she can navigate those services now. But at the beginning, yes, I went to see doctor with her … That’s why, I went to the diabetes clinic. But later on because she can manage now.*

*F 3: That’s a perfect term for it. You [brokers] help us navigate through the system, because without the MCHB we wouldn’t know some of the things that we know now.” (Focus group 2)*

*“They [MCHB] have a lot of programs about culture and about how to … how to fix problem in our home. When we came first time in Canada, we don’t know nothing about Canada. and they fix [resolve] things about our culture and other culture [Canadian culture]… For example, because I don’t know about Canada life when I came first, they translate for me the English and the life how to … how to plan everything. (I6)*

The brokers contribute to helping participants orient themselves in their new lives in Canada.

## Discussion

This study illustrates how the pre- and post migration stressors are entangled with the socio-cultural and economic contexts of refugees and immigrants to present added challenges to the lived experience of diabetes and obesity. The synergistic effects of immigration pathway and its attendant realities, post-immigration stressors of financial constraints, cultural distance and lack of social support presents significant burdens to immigrants and refugees trying to manage their chronic diseases. Thus, although immigrant and non-immigrant patients may struggle with treatment costs, overwhelmed household budgets and limited capacity for lifestyle changes, immigrants have the added burden to first navigate the challenges associated with immigrating and settling into a new environment in order to have the capacity to manage their chronic diseases. Consequently, immigrants and refugees living with diabetes and/or obesity need healthcare that is holistic, whereby it addresses not only their chronic diseases but is able to direct them to or provide them with supports to mitigate the effects of their contextual stressors on their health. Additionally, when healthcare providers are aware of and act in consideration of the patients’ context, it helps to make the care process more meaningful to the patients. We also identified that the work of the brokers contributes to mitigating the effects of the socio-cultural context on the experience of healthcare.

As illustrated in this work, migrants’ ability to manage their chronic conditions is impacted by multi-faceted stressors arising from their pre-and post- immigration realities, including a misalignment of formal systems and services with the realities and needs of migrants, and lack of understanding of migrants’ life contexts. As Shippee and colleagues’ (2012) explain in their cumulative complexity model, these stressors cause a workload-capacity imbalance in chronic disease management. Workload encompasses the demands on the patient’s time and energy, including demands of treatment, self-care, and life in general; and capacity refers to ability to handle work (functional morbidity, financial/social resources, literacy) [22]. Patient workload and capacity are intertwined such that each affects the other; and in situations of imbalance, clinical and social factors accumulate and interact to hinder access, utilization, self-care and health. In relation to our study, newcomers to Canada, people need to make sense of and build new relationships to their environment, including communities, services, built environment, food, cultural ways of interacting and communicating to be able to settle, feel like they belong and to build a livelihood for themselves and their families [23]. This adds to the workload they have to care for their health. The multilevel syndemic interactions of their socio-cultural context affects demand and capacity to manage their chronic disease.

Addressing the impacts of the syndemic interactions of the socio-cultural context and health issues migrants face would require identifying meaningful and impactful ways to help immigrants orient themselves into the society.

For this process, there is a need to develop bidirectional cultural understanding [6] whereby newcomers are supported to understand and navigate the Canadian system, while providers learn to understand and engage with newcomers. Principles of cultural brokering are relevant in this regard as they encompass: 1) mobilizing knowledge of systems and people bidirectionally; 2) mediating and building trust by clarifying misunderstandings on both sides of systems and people to reduce cultural distance; and 3) moving from deeper knowledge to make change through action, through which both the individual and the system can be transformed [24]. Partnerships between healthcare providers and cultural brokers could also help leverage community strengths and resources to address some of the socio-cultural needs and help mitigate the challenges people face in managing their chronic conditions in ways that are meaningful to the members of the ethnocultural communities. Additionally, supports for collaborative and personalized care conversations to find solutions for minimizing patient burdens [25, 26] could improve communication, patient experiences, and self-care, and uncover otherwise-missed opportunities within the limited clinical encounter timeframe [22].

Limitations of this study include the fact that we engaged primarily with community members who utilize the services of the MCHB in Edmonton, and that we were constrained to one interview per person to minimize participant burden. Future in-depth examinations are needed on the challenges immigrant and refugee communities face by immigration pathway. This will improve understanding of the nature of the issues associated with the various immigration routes and may lead to more targeted and effective community-based and healthcare system-based interventions.

## Conclusion

The syndemic effects of the immigrant experience – migration pathway, poverty, cultural distance and lack of social support presents an additional challenge for managing the complexities of diabetes and obesity. This is because people need to first navigate the challenges of being a refugee or immigrant and settling in the new environment in order to have the capacity to manage their chronic diseases. This creates an added burden that can result in inadequate healthcare and worsened health outcomes. Lack of understanding of pre-migration factors, immigration route/status, and post-immigration stressors culminates in a gap in support for diabetes and obesity care. Consequently, care for people with diabetes and/or obesity from vulnerable immigrant and refugee situations should include a holistic approach where there is an awareness of and consideration for their context.

## Data Availability

The qualitative datasets generated and analysed during the current study are not publicly available due to reasons of sensitivity with the data in its raw form, but are available from the corresponding author upon reasonable request.

## References

[1] Brunton S (2016). Pathophysiology of type 2 diabetes: the evolution of our understanding. J Fam Pract.

[2] Wharton S, Lau DCW, Vallis M, Sharma AM, Biertho L, Campbell-Scherer D (2020). Obesity in adults: a clinical practice guideline. CMAJ..

[3] Campbell-Scherer D, Walji S, Kemp A, Piccinini-Vallis H (2020). Canadian adult obesity clinical practice guidelines: primary care and primary healthcare in obesity management.

[4] Luig T, Anderson R, Sharma AM, Campbell-Scherer DL (2018). Personalizing obesity assessment and care planning in primary care: patient experience and outcomes in everyday life and health. Clin Obes.

[5] Spitzer DL. Engendering migrant health: Canadian perspectives. Toronto: University of Toronto Press, Scholarly Publishing Division; 2011.

[6] Ahmed S, Shommu N, Rumana N, Barron G, Wicklum S, Turin T (2015). Barriers to access of primary healthcare by immigrant populations in Canada: a literature review. J Immigr Minor Health.

[7] Shommu NS, Ahmed S, Rumana N, Barron GRS, McBrien KA, Turin TC. What is the scope of improving immigrant and ethnic minority healthcare using community navigators: A systematic scoping review. Int J Equity Health. 2016;15(1). Available from: http://www.equityhealthj.com/content/15/1/6 [cited 2019 Jun 27] 10.1186/s12939-016-0298-8 PMC4714538 26768130

[8] The Multicultural Health Brokers Co-operative. Multicultural Health Brokers | Multilingual, Culturally Diverse Services for Immigrants and Refugees. Available from: http://mchb.org/. [cited 2020 Dec 27]

[9] Hart L, Horton R (2017). Syndemics: committing to a healthier future. Lancet.

[10] Tsai AC, Mendenhall E, Trostle JA, Kawachi I (2017). Co-occurring epidemics, syndemics, and population health. Lancet.

[11] Public Health Agency of Canada. What is the Population Health Approach?. Government of Canada. 2012. Available from: https://www.canada.ca/en/public-health/services/health-promotion/population-health/population-health-approach.html [cited 2021 Mar 9]

[12] Easton D, Ember C, Ember M (2004). The urban poor. Encyclopedia of medical anthropology.

[13] Wallerstein NB, Duran B (2006). Using community-based participatory research to address health disparities. Health Promot Pract.

[14] Israel B, Schulz A, Parker E, Beck A, Allen A, Guzman R, Minkler M, Wallerstein N (2003). Critical issues in developing and following community based participatory research principles. Community based participatory research for health.

[15] Braun V, Clarke V (2006). Using thematic analysis in psychology. Qual Res Psychol.

[16] Castleberry A, Nolen A (2018). Thematic analysis of qualitative research data: is it as easy as it sounds?. Curr Pharm Teach Learn.

[17] Maxwell JA (1992). Understanding and validity in qualitative research. Harv Educ Rev.

[18] Sandelowski M (2000). Whatever happened to qualitative description?. Res Nurs Health.

[19] Chafe R (2017). The value of qualitative description in health services and policy research. Healthc Policy.

[20] Morse JM, Barrett M, Mayan M, Olson K, Spiers J (2002). Verification strategies for establishing reliability and validity in qualitative research. Int J Qual Methods.

[21] Mayan MJ (2009). Essentials of qualitative inquiry.

[22] Shippee ND, Shah ND, May CR, Mair FS, Montori VM (2012). Cumulative complexity: a functional, patient-centered model of patient complexity can improve research and practice. J Clin Epidemiol.

[23] Huttunen L (2010). Emplacement through family life: transformations of intimate relations. COMCAD Arbeitspapiere - Working Papers.

[24] Jezewski MA (1990). Culture brokering in migrant farmworker health care. West J Nurs Res.

[25] Corser W, Holmes-Rovner M, Lein C, Gossain V (2007). A shared decision-making primary care intervention for type 2 diabetes. Diabetes Educ.

[26] Noël G, Luig T, Heatherington M, Campbell-Scherer D (2018). Developing tools to support patients and healthcare providers when in conversation about obesity: the 5As team program. IDJ..

